# Proceedings of the 2015 MidSouth Computational Biology and Bioinformatics Society (MCBIOS) Conference

**DOI:** 10.1186/1471-2105-16-S13-S1

**Published:** 2015-09-25

**Authors:** Jonathan D Wren, Shraddha Thakkar, Ramin Homayouni, Donald J Johann, Mikhail G Dozmorov

**Affiliations:** 1 Arthritis and Clinical Immunology Research Program, Oklahoma Medical Research Foundation; 825 N.E. 13th Street, Oklahoma City, OK 73104-5005, USA; 2 Biochemistry and Molecular Biology Dept, University of Oklahoma Health Sciences Center, USA; 3 Stephenson Cancer Center, University of Oklahoma Health Sciences Center, USA; 4 Department of Geriatric Medicine, University of Oklahoma Health Sciences Center, USA; 5 Division of Bioinformatics and Biostatistics, National Center for Toxicological Research, U.S. Food and Drug Administration, Jefferson, Arkansas, USA; 6 Department of Biological Sciences, University of Memphis, Memphis, TN, USA; 7 Bioinformatics Program, University of Memphis, Memphis, TN, USA; 8 Universtiy of Arkansas for Medical Sciences, Myeloma Institute, USA; 9 Department of Biomedical Informatics, Little Rock, AR, USA; 10 Virginia Commonwealth University, Richmond Academy of Medicine, Department of Biostatistics, USA

## Introduction

The MidSouth Computational Biology and Bioinformatics Society held its twelfth annual conference at the Little Rock Downtown Marriott and Statehouse Convention Center in Little Rock, Arkansas on March 13-14, 2015 (MCBIOS 2015). This year's theme was "*Emerging Trends in Bioinformatics*". The President and Conference Co-Chair this year was Cesar M. Compadre who is a Professor in the Department of Pharmaceutical Sciences at the University of Arkansas for Medical Sciences (UAMS). The other co-chair was William Slikker Jr., the director of the FDA's National Center for Toxicological Research. Conference committee members were Elizabeth Pierce, Shraddha Thakkar, Dennis Burian, Roger Perkins, Weida Tong, Mary Yang, Shweta S. Chawan, Ping Fong Jr., Galina Glazko, Mihir Jaiswal, and Paola Ordonez. Shraddha Thakkar was chosen as President-Elect for 2016-7. There were 158 registrants and a total of 141 abstracts submitted (60 oral and 81 poster presentations).

Keynote speakers were: Carolina Cruz-Neira, the Director of the Emerging Analytics Center at the University of Arkansas at Little Rock (title: "The Pretty Picture: Experiencing Data Through interactive Visual Explorations"); Yana Bromberg, Assistant Professor at the Department of Biochemistry and Microbiology, Rutgers University (title: "Interpreting genomic data to inform pathogenesis pathways"); and Jacque Fetrow, the Provost and Vice President of Academic Affairs at the University of Richmond (title: "What do all those proteins do? An approach to functionally relevant clustering the protein universe").

Participants also had the opportunity to attend a special workshop on "omics in bioinformatics". The presenters in the workshop were Thomas Girke, Director of the Bioinformatics Facility in the Institute of Integrative Genome Biology at University of California, Riverside (title: "Computational Genomics"); Alexey I. Nesvizhskii, Associate Professor in the Department of Computational Medicine & Bioinformatics and Pathology at the University of Michigan, Ann Arbor (title: "Computational Proteomics"); and Frank Emmert-Streib, Associate Professor at the Center for Cancer Research and Cell Biology, Queen's University Belfast (title: "Computational Transcriptomics"). Participants also had chance to attend pre-conference workshop on "R and Bioconductor" by Dr. Girke.

The winners of conference awards were:

**Best Paper Award**: "Efficient experimental design for uncertainty reduction in gene regulatory networks" by Roozbeh Dehghannasiri, Byung-Jun Yoon and Edward Dougherty [1]

Best Oral Presentations (Post-Doctoral fellows):

William S. Sanders, Ph.D., Mississippi State University

Daniel Mohsenizadeh, Ph.D., Texas A&M University

Hui Wen Ng, Ph.D., National Center for Toxicology Research, FDA

Best Oral Presentations (students):

Badri Adhikari, University of Missouri

Zhaofang Li, Rush College

Lauren Bright, Mississippi State University

Best Poster (Computation):

Mihir Jaiswal, University of Arkansas at Little Rock- 1^st ^Place

Jordyn Radke, University of Arkansas at Little Rock- 2^nd ^Place

Jundi Wang, Southern Mississippi University- 3^rd ^Place

Best Poster (Biology):

Shuneize Slater(Lowe), University of Mississippi- 1^st ^Place

Lucky Ahmed, Jackson State University- 2^nd ^Place

Lisa Alley, University of Arkansas for Medical Sciences- 3^rd ^Place

## Selecting papers for the MCBIOS XI proceedings

This year, 20 papers were submitted, and 12 papers were deemed acceptable by reviewers, giving an acceptance rate of 60%. All papers were anonymously peer-reviewed by at least 2 reviewers and quantitatively ranked on the basis of three evaluation criteria: Novelty (1-5), Impact (1-5) and Clarity (1-3). Editors that were co-authors of submitted papers were not permitted to handle their own papers editorially. Papers generally fell into three categories:

### Networks and pathways

Dehghannasiri et al won this year's best paper award for their paper on a method to reduce uncertainty in gene regulatory networks [1] (GRNs). They accomplish this by proposing a quantification of the mean objective cost of uncertainty so that experiments with the least uncertainty become the most highly prioritized. They show on simulated and real GRNs that their method is close to the optimal method, but at a lower cost. Because the computational complexity of analyzing potential GRN structures becomes increasingly burdensome for each new node added, methods to speed up computation enable more accurate approximations.

Mohsenizadeh and colleagues [2] presented a novel methodology to address the dynamic modeling of genomic networks containing uncertainty due to incomplete data. The approach can utilize interaction knowledge provided in public databases. This allows for the construction and study of dynamic interaction-based networks that may be based on small sets of available genomic experimental data.

Huang *et al *report DMAP [3], an extension of the connectivity map (CMAP) concept to identify similar transcriptional effects of drugs upon cell lines. DMAP incorporates additional data on drug to protein effects and effect scores. Using the additional data, DMAP showed increased performance in predicting drug similarity.

Luo *et al *predicted the binding of peptide with Human Leukocyte antigen (HLA) using network analysis [4]. This analysis was performed to generate more understanding of adverse drug reactions (ADR) experience by some patients after taking some drugs. To understand ADR they coupled the network analysis with Nebula to predict the binding interaction of peptides with HLA.

Kim et al identified the genetic subnetwork modules related to maize defense response to the fungal pathogen [5]. This analysis was conducted to understand genetic basis of host (maize) and pathogen (*F. verticilloides*) interaction. They used the network based to approach to identify the maize defense subnetwork activated in presence of the fungus pathogen *F. verticilloides*.

### Genomics & transcriptomics

Peterson *et al *present improvements to their CloneViz software [6] to analyze cancer clonality [7], taking into account multiple types of data gathered, such as whole exome sequencing, RNA-seq and methylation status. They use CloneViz to conduct an analysis of multiple myeloma samples, comparing biopsies gathered at presentation and relapse, finding an amplification of a MYC oncogene with a missense mutation.

Dozmorov *et al*, [8] present a paper concerning an interpretation of the effects from adapter trimming, removal of duplicates, and filtering of low complexity regions. The results show that all the three processing steps will improve the data analysis in RNA-seq and ChIP-seq data in terms of biological signals obtainable by enrichment analysis.

The manuscript by Dohrmann *et al*.[9] addresses and important issue of multiple network alignment, which is broadly useful for comparing pairwise networks derived from different sources. The authors developed a Scaffold-based Multiple Network Aligner (SMAL), which uses a star-like alignment topology and does not rely on native multiple network alignment implementations. They tested SMAL using protein interaction networks from eight different species and showed that the quality of the alignments were comparable to native MNA methods but with much faster performance. The speed and flexibility of SMAL make it ideal for comparison of multiple large networks derived from an expanding array of omics platforms.

### Miscellaneous

Automated high resolution dermoscopic image analysis techniques can be very useful in clinical settings by elucidating information that would otherwise be unnoticeable. Lemon and colleagues [10] parallelized a prominent skin lesion border detection algorithm by using the new WebCL web browser based parallel programming technology on graphical processing units (GPUs). The parallel version of the border detection algorithm had the same accuracy with 4-5 fold faster speeds and provides the flexibility to run on mobile devices, making it ideal for use in clinical settings.

Lencioni *et al *demonstrated the adverse event (AE) capture management software for cancer studies. [11]. They created this to help with decisions crucial for clinical trials. They used the standards based AE Reporting System (AERS) in integration with Epic Electronic Health Record for tracking the current and recent AEs. This system is currently to supporting the 350 patients enrolled for around 65 different cancer studies.

In the paper by Zhao and colleagues [12], the authors presented a heuristic method called Rate of Perplexity change (RPC) to determine the optimal number of topics in a collection of text documents after applying the well-known Latent Dirichlet Allocation (LDA) method. The RPC method was evaluated on three widely different data types and sizes and was found to be stable, accurate and effective.

Madahian *et al *[13] report a new classification method and applied it to a microarray gene expression dataset with comparisons to other classification methods. The approach employed a Generalized Double Pareto (GDP) prior to induce sparsity in a Bayesian Generalized Linear Model and resulted in an improved prostate cancer subclass prediction especially with pre-metastatic stages.

## Future meetings

Approaches towards applied biomedical science, basic research and clinical medicine are rapidly changing due to next generation sequencing (NGS). This is especially true for cancer-based endeavors and the practice of clinical oncology. President Obama's Precision Medicine Initiative calls for a near-term focus on cancers and a longer-term aim to generate knowledge applicable to a whole range of health and disease [14]. Why now? Both goals are within reach due to i) advances in genomics over the past 10 years, ii) increasing use of electronic health records, iii) technical advances in health devices in smart phones and the fact a majority of US adults own one, iv) advances in data science especially concerning big data, and v) the changing role of patient partnerships especially crowd sourcing and citizen science, where people want to participate with feedback. Since the cost of a Whole Exome Sequencing (WES) study is ~$1000 (the price of a CT scan), NGS assays are expected to proliferate and become the standard of care in the future. Implementation of precision medicine into the clinical workflow requires significant changes in infrastructure, work force development and logistics. The vital infrastructure involves advanced analytics including decision support, information technology and high-performance computing capabilities. A precision medicine clinic would have to seamlessly integrate patient genetic counseling, sample collection, storage, sequencing, analysis and secure delivery of genomic-based information to the physician at the point of care [15]. So, what exactly is precision medicine?

Precision medicine is an innovative approach that takes into account individual differences in people's genes, environments, and lifestyles. It gives medical professionals the resources they need to target treatments, improve outcomes, and reduce side effects. In oncology, the ultimate aim of precision medicine is to keep people healthier by detecting cancer earlier, using an individual (versus categorical) approach for therapeutic assignments, and reducing therapeutic related toxicities. Cancer precision medicine uses genetic information from a patient's tumor to determine a rationally derived therapy plan targeted to the particular genetic abnormality. It is also furthering the study of correlations and associations of cancer patients in terms of: i) genotype to phenotype and ii) phenotype to genotype. By using individual genetic information to prevent, diagnose earlier, and treat disease/cancer with better precision, this type of genomics-enabled medicine promises health care that is personalized, more predictive, and preventive rather than reactive. In summary, precision medicine targets the needs of individual patients on the basis of genetic, biomarker, phenotypic, or psychosocial characteristics that customize care. Precision medicine approaches will be judged by rigorous evaluation of "providing value" via analyses of i) efficacy, ii) safety, and iii) cost effectiveness [16]. Please join us for MCBIOS 2016 from March 3^rd^-5^th ^in Memphis, Tennessee where precision medicine will be the conference theme.

## Competing interests

The authors declare that they have no competing interests.

## Authors' contributions

All authors of this paper served as editors for these proceedings, with JDW serving as Senior Editor. All authors helped write this editorial.
